# Comorbidities and Risk Factors of Patients Diagnosed with CRC after Positive Fecal Test in Real Life

**DOI:** 10.3390/cancers14225557

**Published:** 2022-11-12

**Authors:** Naim Abu-Freha, Rachel Gouldner, Bracha Cohen, Michal Gordon, Orly Sagi, Gadeer Taha, Liza Ben Shoshan, Zohar Levi

**Affiliations:** 1 The Institute of Gastroenterology and Hepatology, Soroka University Medical Center, Beer-Sheva 84105, Israel; 2 The Faculty of Health Sciences, Ben-Gurion University of the Negev, Beer-Sheva 84105, Israel; 3 Medical School for International Health, Ben-Gurion University of the Negev, Beer-Sheva 84101, Israel; 4 Soroka Clinical Research Center, Soroka University Medical Center, Beer-Sheva 84101, Israel; 5 Bacteriology Laboratory, Soroka University Medical Center, Beer-Sheva 84101, Israel; 6 Department of Gastroenterology, Rambam Health Care Campus, Haifa 31096, Israel; 7 Department of Gastroenterology, Beilinson Medical Center, Sackler School of Medicine, Tel Aviv University, Tel Aviv 39100, Israel

**Keywords:** fecal testing, colorectal cancer, screening, risk factor

## Abstract

**Simple Summary:**

Occult blood in stool is the screening modality of choice for colorectal cancer in most countries. Only a small percentage of patients with positive results of this test will have colorectal cancer. In the present study, we wanted to determine risk factors for having colorectal cancer among patients with positive fecal testing. We found that age, anemia, family history of colorectal cancer, and previous colorectal cancer are factors for having colorectal cancer among those with positive fecal testing. The practical implication of the findings of the present study is that people who have positive fecal testing and one of the found risk factors should be prioritized for colonoscopy scheduling.

**Abstract:**

(1) Background: Fecal occult blood test (FOBT) is the modality of choice in most countries for colorectal cancer (CRC) screening. We aimed to investigate the risk factors for CRC among patients with a positive FOBT in real life. (2) Methods: This was a retrospective study that included patients who tested positive for FOBT. Data regarding the comorbidities and laboratories were collected and compared between CRC and non-CRC groups. (3) Results: Positive FOBT was found among 45,500 (5.36%) subjects and CRC was found in 1502 (3.3%). CRC patients were older, age 62.7 ± 7.15 years compared with 59.33 ± 7.3 years (*p* < 0.001), and had significantly higher rates of hypertension (48.4% vs. 44.7%, *p* = 0.002), iron-deficiency anemia (20.6% vs. 16.4, *p* < 0.001), family history of CRC (7.3% vs. 5.1%, *p* < 0.001), and previous CRC (6.5% vs. 0.3%, *p* < 0.001). Lower levels of hemoglobin, iron, and ferritin were found in the CRC group. Age, family history of CRC, and previous CRC were found to be significant risk factors for diagnosis of CRC after positive FOBT with OR of 1.057, 1.4, and 15.9, respectively. (4) Conclusions: Iron-deficiency anemia, family history of CRC, previous colorectal cancer, and low hemoglobin, iron, and ferritin levels should direct physicians to give high priority to colonoscopy scheduling.

## 1. Introduction

Colorectal cancer (CRC) is a common cancer, ranked as the third-most-common cancer worldwide, but as the second cancer in terms of mortality [1]. Wide geographical variation is observed in CRC incidences, with the highest rates being found in European regions, Australia/New Zealand, and Northern America, with Hungary and Norway ranking first in men and women [1]. The GLOBOCAN data estimated more than 1.9 million new colorectal cancer (including anus) cases and 935,000 deaths were estimated to occur in 2020, representing about 1 in 10 cancer cases and deaths [1]. CRC, with its high incidence rate, the long preclinical phase, recognizable and tractable precursor, and the correlation between the tumor stage and mortality rate, fulfills the criteria of Wilson and Jungner as the ideal disease for screening [2]. Several different modalities are used worldwide for CRC screening, mostly among the population aged between 50 and 75 years. An annual or biennial fecal immune chemical test (FIT) or colonoscopy every 10 years is the recommended modality in most countries. Additional methods include flexible sigmoidoscopy, multitarget stool DNA test, virtual colonoscopy (computer tomography colonography), or colon capsule, which could be suggested [3,4]. However, significant differences and disparities in implementation strategies are found in the different countries [4]. Participation in screening is still an important challenge in many countries. The Netherlands showed the highest participation rate (68.2%) and some areas of Canada showed the lowest (16%). Participation rates were highest among women and in programs that used the FIT test. Men exhibited the greatest number of positive results [4].

A fecal occult blood test (FOBT) is the stool-based screening modality of choice for colorectal cancer (CRC) in most countries. The goal of CRC screening is the early detection of colon neoplasia in terms of advanced adenoma or carcinoma. As a result of CRC screening, early detection, removal of adenomas, and a reduction in mortality can reached [5,6]. Both the removal of precancerous adenomatous polyps at the time of colonoscopy and early detection of curable CRC have been shown to reduce CRC mortality [7]. On one hand, CRC is the most feared abnormal finding that can be found in the colonoscopy performed after positive FOBT but, on the other hand, only a small proportion of patients with positive FOBT are diagnosed with CRC. In a previous study performed in Israel, 3.38% of people with positive FOBT had a diagnosis of CRC [8]. In another study, a CRC diagnosis among 2.98% of the 81,518 positive FOBTs was found [9]. Not all those with positive FOBTs undergo colonoscopy, as about 10–33% of positive FOBT patients elect not to [10,11,12,13,14,15,16]. It is important to try to differentiate high-risk patients for CRC after having a positive FOBT test. Additionally, sources are limited and most patients with a positive FOBT will have normal or non-significant findings. To save resources, it is important to single out high-risk patients for CRC or polyps and put our focus on these patients for future interventions and to bring them in for colonoscopy, particularly in the post-COVID-19 era with all the impact related to the pandemic on screening programs and cancers diagnosis. Prioritization of patients with positive FOBT in general and particularly among those who have a high risk for CRC could have an enormous impact on the disease stage at the time of diagnosis and, as a result, the need for additional therapies and mortality. In Israel, fecal immunochemical testing (FIT) is available for populations aged 50–74 years. Patients who have a positive FIT are recommended to perform a colonoscopy. In 2019, data in Israel showed that 65.4% of the target population performed FOBT in the previous years or colonoscopy during the 10 years before [17]. In general, most people undergo the FOBT test; however, a small number of patients could choose colonoscopy as the modality for CRC screening rather than FOBT. Patients with a previous diagnosis of CRC are recommended to undergo colonoscopic surveillance and not perform FOBT according to the known guidelines. Until now, there have been no data published regarding risk factors for CRC among positive FOBT patients in real life. In the present study, we aimed to investigate the risk factors in terms of comorbidities and laboratories of patients with positive FOBT diagnosed with CRC compared to those with positive FOBT but negative for CRC.

## 2. Materials and Methods

### 2.1. Study Population

In the present study, we enrolled subjects who performed fecal testing as a part of the CRC screening program between the ages 50 and 74 years and have positive FOBT tests during the study period, between the dates 1 January 2010 and 30 December 2020. An outreach-based annual fecal occult blood test screening program operated by Israel’s Health Maintenance Organizations (HMOs) was conducted. Since June 2016 FIT stool testing has been used for CRC screening while guaiac-based testing was used before. The study population was divided into two groups: the CRC group—subjects with positive FOBT and diagnosed with CRC—and the Non-CRC group—those with positive FOBT, but not diagnosed with CRC. CRC was considered to be related to the same FOBT test until two years after a positive test; patients diagnosed with CRC more than two years after the FOBT were excluded. CRC diagnosis was measured until two years following the performance of FOBT. In cases of positive FOBT without CRC, a follow-up was continued during the study period, while patients diagnosed with CRC were not followed after the CRC diagnosis.

### 2.2. Data Collection

The data were collected from the organized CRC screening program for both study groups. Demographic (age, gender) clinical and laboratory data were retrospectively collected, using data and results closest to performing the FOBT test. The number of entire performed tests, positive tests, and CRC diagnoses was collected. In addition, laboratory data including complete blood count (CBC), biochemistry, and Carcinoembryonic antigen (CEA) were also collected. The laboratory values were collected nearest to the fecal occult blood testing. Comorbidities (chronic ischemic heart disease, congestive heart disease, diabetes mellitus, chronic obstructive lung disease, and etc.) of the enrolled patients were reviewed and compared between the two groups. No data were available regarding adenoma or advanced adenoma found in the colonoscopy.

### 2.3. Data Sources

The data were extracted from Clalit Health Services (CHS) using Clalit’s Data sharing platform powered by MDclone (https://www.mdclone.com, accessed on 11 May 2022). The MDclone platform includes synthetic data of patients of the HMO to protect the patient’s privacy. The platform includes data of every patient including diagnosis, events such as hospitalization, emergency department visits, surgeries, and laboratory results. Any new diagnosis, hospitalizations, or medical updates considered as new events for the specific patients can be extracted in relation to the lifespan of the patient. CHS is the largest of the four health maintenance organizations in Israel, with about 4.7 million insured, accounting for 53% of the Israeli population, and considered as one of the large HMOs worldwide.

### 2.4. Statistical Analysis

Data are presented as a mean ±SD for continuous variables and as a percentage (%) of the total for categorical variables. Univariate analyses were performed by the Mann–Whitney test (for continuous variables) and Fisher’s exact and chi-squared tests (for categorical variables). All statistical analyses were performed using IBM SPSS version 26 (Chicago, IL, USA). *p*-values less than 0.05 were considered statistically significant. Before introducing the variables into the model, the multicollinearity of the variables was examined, using the Variance Inflation Factor (VIF) statistic. Variables, which could impact or be considered as risk factors such as age, gender, diabetes mellitus, iron-deficiency anemia, smoking, family history of CRC, or previous CRC were included in the model. The variables found to be significant in the univariate analysis were introduced into the multivariate model one after the other and non-significant variables in the univariate model were not included in the multivariate analysis. The order in which the variables entered the model was determined by the size of the univariate Odds Ratio. A logistic regression model was used for the prediction of risk factors for CRC diagnosis, age, gender, diabetes mellitus, iron-deficiency anemia, smoking, family history of CRC, and previous CRC were included in the model. The study was carried out in accordance with the principles of the Helsinki Declaration, approval number 0093-21-SOR of the local Helsinki committee in Soroka University Medical Center. The study was waived from informed consent due to the retrospective nature of the study.

## 3. Results

### 3.1. Patients

During the study period, 847,550 FOBTs were performed and 45,500 (5.36%) of the tests were positive. All patients are in the age group of CRC screening, between 50 and 74 years. During the long study period, two types of FOBTs were used in Israel, guaiac and fecal immunochemical (FIT) tests. Starting in 2016, the FIT test is used, while before 2016, the guaiac was the test used for screening. In our study, there were 25,372 guaiac-based tests (55.8%) and 20,128 (44.2%) FIT-based tests. Among those with positive tests, 1502 (3.3%) patients were diagnosed with colorectal cancer after the FOBT. The basic characteristics, demographics, and comorbidities of the two study groups (CRC and non-CRC groups) are summarized in Table 1. Patients who were diagnosed with CRC were found to be significantly older, aged 62.7 ± 7.15 years, compared to 59.33 ± 7.3 years for those without a CRC diagnosis (*p* < 0.001). In addition, patients diagnosed with CRC were more often found to have hypertension (48.4% vs. 44.2%, *p* = 0.002), a family history of CRC (7.3% vs. 5.1%, *p* < 0.001), and iron-deficiency anemia (20.6% vs. 16.4%, *p* < 0.001) as comorbidities. No significant differences were found regarding other comorbidities (chronic ischemic heart disease, congestive heart failure, chronic obstructive lung disease, chronic kidney disease…). Patients diagnosed in the past with CRC along with a positive FOBT had significantly higher CRC recurrence than those with a negative FOBT (9.6% vs. 0.3%, *p* < 0.001). No significant difference regarding gender was found (the male proportion was 52.3% among the CRC group compared to 53.9% in the non-CRC group, *p* = 0.220). In addition, there was no significant difference regarding body mass index (BMI) but a lower rate of smoking in the CRC groups, 30% vs. 33.5%, *p* = 0.007.

### 3.2. Laboratory Values

The laboratory values nearest to the time of FOBT of both groups were collected and are presented in Table 2. Several significant differences between the CRC and non-CRC groups were found; patients diagnosed with CRC after positive FOBT were found to have lower hemoglobin levels (12.8 ± 1.94 vs. 13.64 ± 1.7, *p* < 0.001), albumin (4.14 ± 1.5 vs. 4.23 ± 1.3, *p* = 0.018), iron (62.69 ± 174 vs. 76.7 ± 34, *p* < 0.001), and ferritin levels (101.36 ± 174 vs. 123.43 ± 38, *p* < 0.001). In addition, a higher count of platelets, 275 ± 90 vs. 250 ± 74, *p* < 0.001) and a higher CEA level (64.83 ± 569 vs. 12.17 ± 274, *p* < 0.001) were also observed among the CRC group. No significant differences were found regarding most other laboratory values (WBC, AST, GGT, and etc.).

### 3.3. Risk Factors for CRC

Uni- and multivariable analysis models for CRC diagnosis after positive FOBT were calculated and the results of the model for CRC diagnosis are presented in Table 3. In the multivariable analysis, age 1.057, *p* < 0.001, family history of CRC OR 1.4, *p* = 0.001, and previous CRC OR 15.9, *p* < 0.001 were found to be significant risk factors for CRC diagnosis among patients with positive FOBT. In the univariable model, the age (OR 1.063, *p* < 0.001), iron-deficiency anemia (OR 1.318, *p* < 0.001), family history of CRC (OR 1.471, *p* < 0.001, and previous CRC (OR 22.433, *p* < 0.001) were found to be risk factors for CRC. Diabetes mellitus and gender were not found to be significant risk factors. In the multivariate analysis model, we observed an OR of 1.057 for age (*p* < 0.01), OR of 1.131 (*p* < 0.072) for iron-deficiency anemia, OR of 1.0404 for family history of CRC (*p* < 0.001), and OR of 15.935 (*p* < 0.001) for previous CRC.

## 4. Discussion

In this study, we evaluated the rate of CRC among patients with positive FOBT within an organized population-based CRC screening program and factors associated with increased risk for CRC. Overall, we report that about 3.3% of all patients with positive FOBT were diagnosed eventually with CRC. The main findings of our study are that subjects with a previous history of CRC, family history of CRC, iron-deficiency anemia, or lab values of anemia (low hemoglobin, iron, and ferritin) were at significantly increased risk for harboring cancer. This finding is novel and has not been reported before in the context of real-life data until now and with a such large number of patients. Interestingly, subjects with either previous CRC or a family history of CRC or anemia should have been referred for colonoscopy and not FOBT. A significant proportion of the total subjects with positive FOBT have one of these indications, making up about 35% of all subjects with positive FOBT in our study. However, our data showed that in real life, practically, not all people who have a clear indication for colonoscopy will undergo it; instead, they perform stool testing. There are several reasons behind not completing a colonoscopy; however, only scant data has been published, focused on adherence to post-CRC surveillance colonoscopy. Metastasis and refusal of the patients for post-CRC surveillance were found to be reasons for nonadherence to colonoscopy among this specific group [18]. In fact, adherence to colonoscopy was intensely researched among patients who had positive FOBT and there are several reasons for barriers to endoscopic follow-up, including the four main groups: information technology (IT) [19,20], healthcare organizations [21,22], physician behavior [23,24,25,26], and patient emotional and cognitive factors [27,28,29,30]. In addition, barriers could affect adherence to colonoscopy and the barriers could be related to the patients, providers, and system barriers [31].

It is important to mention that iron-deficiency anemia is found at a higher rate among those diagnosed with CRC. In the present study, we found a higher rate of iron-deficiency anemia, appearing as “iron-deficiency anemia” in the list of diagnoses. Additionally, the levels of hemoglobin, iron, and ferritin were significantly lower among CRC patients. Of note, the diagnosis of iron-deficiency anemia was found to be a significant risk factor for CRC diagnosis in the univariate model analysis, while only a trend of statistical significance in the multivariate model analysis. Out of this, any patient with a positive FOBT combined with iron-deficiency anemia should be seriously encouraged to undergo a colonoscopy. Previous studies reported a higher rate of CRC among patients with a combination of a positive FOBT and iron-deficiency anemia. CRC was detected in 2.7% of patients with positive FOBT and iron-deficiency anemia compared to 0.4% for patients with a positive FOBT without iron-deficiency anemia [32,33].

An interesting finding is that patients with previous colon cancer have the highest risk for CRC diagnosis after a positive FOBT. Actually, patients with previous colon cancer should not perform FOBT but should perform a follow-up colonoscopy due to their high risk for recurrence disease and additional adenoma of carcinoma. Patients with previous CRC should be scheduled for post-CRC surveillance colonoscopy, according to the known guidelines. However, in real life, as we found in our study, a small selection of the performed FOBTs were conducted among patients with a previous diagnosis of CRC and, as found in our results, these people were found to be at the highest risk for CRC recurrence. Performing FOBT among part of the patients after CRC in our cohort could be related to patients’ preferences or to the fact that the FOBT test is sent automatically annually for patients in the target age group. Additional studies are needed in other countries to understand if it is common in other regions and local studies are needed in order to attain a deep understanding of this point. Despite this finding, we still suggest colonoscopy as a follow-up modality of choice in patients treated for CRC and continued surveillance, but the cases of patients not completing colonoscopy and having positive FOBT should be scheduled as priority for colonoscopy due to their high risk.

The importance of analyzing possible risk factors is to identify high-risk subjects and to encourage them to undergo a colonoscopy. Singling out those with higher risk is of particular importance due to the low adherence to colonoscopy after a positive FOBT with the known diversity between the populations. The colonoscopy completion rate after positive FOBT varies between 30 and 80% [31,34]. Identifying those classified as high risk, scoring them according to their age, comorbidities, and laboratory findings, and intervening specifically among this group of patients with the recommendation that they should conduct a colonoscopy and giving them high priority for procedure scheduling is of great importance.

In general, in the era of aging populations, increasing awareness regarding screening for cancer is a priority and colonoscopy is an important part, beginning at the level of healthcare organizations and providers using information technology. The risk for CRC for every patient with positive FOBT should be calculated. The calculation model should include the age, comorbidities, including diagnosis of iron-deficiency anemia, family history of CRC, and previous CRC, and laboratory values, including hemoglobin, iron, ferritin, and CEA. Patients at high risk should be prioritized for colonoscopy. The CRC risk evaluation can be made by general practitioners using a simple evaluation of the medical file, including the diagnosis list of the patients and evaluating the laboratory values and case of a conclusion that the patients are at high risk for CRC. The next step should be explaining the findings to the patients and their high-risk status in order to increase adherence to colonoscopy and to avoid postponing the colonoscopy. This is crucial as postponement of colonoscopy is associated with more advanced disease and higher mortality due to CRC among patients who performed their colonoscopies more than 12 months after the initial positive fecal occult blood test, as reported before [8].

In summary, in this large study conducted within the Clalit, one of the largest HMOs worldwide, we report that a significant proportion of the subjects with positive FOBT eventually having CRC had iron-deficiency anemia, family history of CRC, or previous CRC. Although these patients should have been referred for colonoscopy primarily, once they have a positive FOBT, they should be prioritized for colonoscopy. Taken in the context of the post-COVID-19 era, with a huge impact on the screening program and a decrease in the endoscopy procedures performed in 2020, the disruption of screening programs in most countries resulted in a decrease in CRC incidence in 2020 compared to 2019 [35]. Moreover, the projected effect of short-term disruption to colorectal cancer screening will have a marked impact on colorectal cancer incidence and deaths between 2020 and 2050, attributable to missed screening [35,36]. Thus, it is crucial to ensure participation rates return to previously observed rates and we suggest a special consideration for patients with risk factors for CRC, as we found in the present study. To reach this goal, active action and interventions should be carried out by healthcare providers and healthcare policymakers.

The strengths of our study are the national-based design, with a large number of enrolled subjects, and access to the computerized medical file, including diagnosis and laboratories. To the best of our knowledge, there is no previous study that investigated this important issue with such a large number of patients and in a real-life setting.

Despite these strengths, there are several limitations to mention. Firstly, the retrospective design, secondly, the long study period included using both the guaiac-based and FIT-based testing in the study, thirdly, no data were available regarding symptoms before colonoscopy or polyp/adenoma detection or localization of the neoplasm, and, lastly, in the platform, there are no accurate data regarding smoking because they are coded within the electronic medical records as different conditions. Smoking could be listed as: current smoker, a current smoker and wants to stop, or past smoker, so it is a weakness of the platform to obtain accurate data for this metric; lastly, not all patients may have complete laboratory data.

## 5. Conclusions

Patients with positive FOBT and iron-deficiency anemia, previous colorectal cancer, family history of CRC, low hemoglobin, iron, and ferritin are at higher risk to be diagnosed with CRC. Finding one of these factors should direct physicians to give high priority to colonoscopy scheduling. These patients should be prioritized other average-risk patients. In addition, high-risk patients with a strong indication for colonoscopy should be referred for colonoscopy and not for fecal occult blood stool testing.

## Figures and Tables

**Table 1:** Demographics and comorbidities in the study groups.

	CRC after Positive FOBT *n* = 1502 (%)	No CRC after Positive FOBT *n* = 43,998 (%)	*p*-Value
Guaiac based FOBT	1043 (69.4)	24,329 (55.3)	<0.001
FIT based FOBT	456 (30.6)	19,669 (44.7)	<0.001
Gender (male)	785 (52.3)	23,701 (53.9)	0.220
Age (years, mean ± SD)	62.7 ± 7.15	59.33 ± 7.3	<0.001
BMI	28.6 ± 5.8	28.6 ± 6	0.998
Smoking	446 (30)	14,555 (33.5)	0.007
Family history of CRC	110 (7.3)	2243 (5.1)	<0.001
Previous CRC	97 (6.5)	135 (0.3)	<0.001
Comorbidities			
CIHD	228 (15.2)	7413 (16.8)	0.089
CHF	44 (2.9)	1433 (3.3)	0.481
COPD	107 (7.1)	3880 (8.8)	0.022
Asthma	130 (8.7)	4448 (10.1)	0.065
CRF	106 (7.1)	2648 (6)	0.097
HTN	733 (48.4)	19,650 (44.7)	0.002
DM	194 (12.9)	5307 (12.1)	0.318
Dyslipidemia	850 (56.5)	24,388 (55.7)	0.474
Obesity	494 (32.9)	15,498 (35.2)	0.062
CVA	32 (2.1)	806 (1.8)	0.397
Dementia	14 (0.9)	277 (0.6)	0.148
Fatty liver	107 (7.1)	3342 (7.6)	0.497
Depression	138 (9.2)	4928 (11.2)	0.015
Vitamin B12 Def.	9 (0.6)	285 (0.6)	0.817
Folic acid def	223 (14.8)	6687 (15.2)	0.709
Iron deficiency A	309 (20.6)	7229 (16.4)	<0.001
Vitamin D def	198 (13.2)	5449 (12.4)	0.356

**Table 2:** Laboratory values of the study groups.

	CRC after Positive FOBT *n* = 1502 (%)	No CRC after Positive FOBT *n* = 43,998 (%)	*p*-Value
HB (gr/dL)	12.8 ± 1.94	13.64 ± 1.7	<0.001
WBC (10^3^/uL)	7.57 ± 2.34	7.48 ± 3.04	0.292
PLT (10^3^/uL)	275 ± 90	250 ± 74	<0.001
AST (U/L)	23.19 ± 22	23.93 ± 21	0.187
ALT (U/L)	21.18 ± 22	23.44 ± 20	<0.001
GGT (U/L)	46.17 ± 111	41.89 ± 84	0.151
Creatinine (mg/dL)	0.87 ± 0.53	0.86 ± 0.56	0.823
Albumin (gr/dL)	4.14 ± 1.5	4.23 ± 1.3	0.018
Vitamin D	48.9 ± 25.1	47.5 ± 24	0.099
Vitamin B12	327.5 ± 167	337.6 ± 180	0.047
Folic acid	19.9 ± 52	18.4 ± 45	0.289
HbA1c (%)	6.29 ± 1.2	6.26 ± 1.3	0.409
Iron (mcg/dL)	62.69 ± 37	76.7 ± 34	<0.001
Ferritin	101.36 ± 174	123.43 ± 308	<0.001
Calcium	9.37 ± 0.53	9.39 ± 0.47	0.121
CEA	64.83 ± 569	12.17 ± 274	<0.001

**Table 3:** Uni- and multivariate A logistic regression model for the association between positive FOBTs and diagnosis of colorectal cancer.

Univariable Analysis				Multivariable Analysis		
	OR	95% CI	*p*-Value	OR	95% CI	*p*-Value
Age at diagnosis	1.063	1.055–1.070	<0.001	1.057	1.050–1.065	<0.001
Gender	0.938	0.846–1.039	0.220	NA	NA	NA
Diabetes Mellitus	1.081	0.928–1.261	0.318	NA	NA	NA
Iron-deficiency Anemia	1.318	1.160–1.497	<0.001	1.131	0.989–1.293	0.072
Smoking	0.857	0.765–0.959	0.007	0.865	0.771–0.970	0.013
Family history of CRC	1.471	1.206–1.794	<0.001	1.404	1.147–1.719	0.001
Previous CRC	22.433	17.190–29.276	<0.001	15.935	12.054–21.064	<0.001

## Data Availability

No additional data are available.
